# Higher Cumulative Cytarabine Consolidation Improves Survival in Older Adults with Acute Myeloid Leukemia

**DOI:** 10.3390/cancers18111831

**Published:** 2026-06-03

**Authors:** Todd William Mudd, Kendall Diebold, Sravanti Rangaraju, Aditi Sharma, Kimo Bachiashvili, Pankit Vachhani, Manuel R. Espinoza-Gutarra, Razan Mohty, Ravi Bhatia, Jorge Cortes, Omer Jamy

**Affiliations:** 1 Department of Medicine, University of Alabama at Birmingham, Birmingham, AL 35294, USA; 2 Division of Hematology/Oncology, Department of Medicine, University of Alabama at Birmingham, Birmingham, AL 35294, USA

**Keywords:** AML, older adults, cytarabine, consolidation

## Abstract

**Background**: Post-remission cytarabine consolidation is a cornerstone of therapy for acute myeloid leukemia (AML), but the optimal dosing strategy in older adults (≥60 years) remains unclear. High-dose cytarabine (HiDAC) is often avoided due to toxicity concerns, and data guiding cumulative dosing are limited. **Methods**: We conducted a single-center retrospective cohort study of 111 patients aged ≥60 years with AML who achieved complete remission after standard 7 + 3 induction and received at least one cycle of cytarabine consolidation between 2012 and 2024. A 90-day landmark analysis excluded early relapses or deaths. **Results**: The median age was 65 years; 41% proceeded to allogeneic hematopoietic stem cell transplantation (allo-SCT). Cytarabine consolidation was well tolerated, with no neurotoxicity and only one instance of reversible nephrotoxicity. Patients were stratified by median cumulative cytarabine dose into low-intensity (<18 g/m^2^, LIC) and high-intensity (≥18 g/m^2^, HIC) groups. HIC was associated with improved overall survival compared with LIC (median OS: 31 vs. 13 months, *p* = 0.02), particularly among non-transplanted patients (25 vs. 7 months, *p* = 0.01). On multivariable analysis, HIC (HR 0.71, 95% CI 0.51–0.82, *p* = 0.01) and allo-SCT (HR 0.58, 95% CI 0.44–0.79, *p* = 0.03) independently predicted superior survival. **Conclusions**: Higher cumulative cytarabine consolidation is safe, feasible, and associated with improved survival in older AML patients, especially among patients ineligible for transplant. Prospective studies are warranted to define the optimal dosing strategy in this population.

## Simple Summary

We investigated whether higher doses of cytarabine consolidation therapy improve outcomes in 111 older adults with acute myeloid leukemia (AML). The treatment was generally safe and well tolerated. No patients developed neurotoxicity, and only one patient experienced reversible kidney toxicity. Patients receiving higher cumulative doses of cytarabine (≥18 g/m^2^) had improved survival than those receiving lower doses. This benefit was especially clear in patients who did not undergo stem cell transplantation. We also found that both higher dose cytarabine and stem cell transplantation independently improved survival. These results suggest that higher cumulative cytarabine dosing may provide meaningful benefit for older AML patients, particularly those who are not transplant candidates.

## 1. Introduction

Post-remission consolidation therapy with cytarabine is a cornerstone of treatment for patients with acute myeloid leukemia (AML) who achieve complete remission following standard 7 + 3 induction chemotherapy (cytarabine for 7 days plus an anthracycline for 3 days) [1]. Early randomized trials, including the CALGB 8525 study, demonstrated that high-dose cytarabine (HiDAC) consolidation significantly improved remission duration and survival outcomes in younger patients with AML [2,3]. As a result, cytarabine-based consolidation became the standard post-remission strategy for patients eligible for intensive therapy [4,5,6,7,8].

More recent studies have evaluated whether intermediate-dose cytarabine (IDAC) provides comparable efficacy to traditional HiDAC consolidation. Several retrospective studies and randomized trials have reported similar overall survival and relapse-free survival outcomes between intermediate- and high-dose cytarabine regimens [9,10,11,12]. Consequently, contemporary treatment guidelines recommend intermediate-dose cytarabine as an acceptable consolidation strategy for most younger patients with AML.

However, the optimal cytarabine consolidation strategy for older adults fit for intensive chemotherapy remains unclear. High-dose cytarabine has historically been avoided in patients aged ≥60 years because of concerns regarding treatment-related toxicity, particularly neurotoxicity and nephrotoxicity [2,3]. As a result, older patients are frequently treated with reduced-dose consolidation regimens, and data guiding the optimal cumulative cytarabine dose in this population are limited.

Given the increasing use of IDAC and the relative lack of prospective data in older patients, there remains an important knowledge gap regarding the most effective consolidation dosing strategy in this population. In particular, the impact of cumulative cytarabine exposure on survival outcomes among older adults with AML has not been well defined. To address this gap, we investigated the outcomes of patients aged ≥60 years with AML who received cytarabine consolidation therapy at our center.

## 2. Methods

### 2.1. Study Design and Patient Population

We conducted a single-center retrospective cohort study of consecutive patients aged ≥60 years diagnosed with non-M3 AML who received cytarabine-based consolidation therapy at our institution between January 2012 and December 2024. This study was approved by our Institutional Review Board, and the requirement for informed consent was waived due to its retrospective design.

Patients were included if they achieved remission following induction chemotherapy and subsequently received at least one cycle of cytarabine consolidation. To minimize bias from early relapse or death and to focus on patients eligible for extended consolidation therapy, a 90-day landmark analysis from the start if induction therapy was applied. Patients who experienced relapse or death before the 90-day landmark were excluded from the analysis. Clinical and demographic data were obtained through retrospective review of the electronic medical record. Baseline disease risk was classified according to the 2022 European LeukemiaNet (ELN) risk stratification criteria. This study specifically evaluated older adults with AML considered fit for intensive chemotherapy and post-remission consolidation. Fitness for intensive therapy was determined retrospectively based on institutional clinical practice, including assessment of performance status, comorbidity burden, organ function, and treating physician judgment at the time of treatment decision-making. Because of the retrospective nature of the study, standardized geriatric assessments and formal comorbidity indices were not uniformly documented or available for analysis. Consequently, patient fitness could not be quantified using validated scoring systems, and treatment intensity decisions may have been influenced by clinician-perceived frailty or comorbidity risk.

### 2.2. Induction and Consolidation Therapy

All patients received standard induction chemotherapy consisting of 7 + 3. The anthracycline administered was either idarubicin 12 mg/m^2^ or daunorubicin 45 mg/m^2^ at the discretion of the treating physician. Cytarabine was administered at 100 mg/m^2^. Patients who did not achieve remission following initial induction therapy were eligible to receive re-induction therapy.

Patients who achieved first complete remission proceeded to cytarabine-based consolidation therapy. Cytarabine was administered according to institutional practice using either a condensed schedule (days 1, 2, and 3) or a standard schedule (days 1, 3, and 5). Individual cytarabine doses ranged from 1 g/m^2^ to 3 g/m^2^ per dose, corresponding to a total dose per cycle between 6 g/m^2^ and 18 g/m^2^. The number of consolidation cycles administered was determined by treating physicians based on clinical judgment, treatment tolerance, and transplant planning.

### 2.3. Statistical Analysis

Baseline patient characteristics were summarized using descriptive statistics. Survival outcomes were estimated using the Kaplan–Meier method and compared between groups using the log-rank test. Subgroup analyses were performed according to consolidation dose intensity, number of consolidation cycles, and receipt of allogeneic hematopoietic stem cell transplantation (allo-SCT).

Univariable and Multivariable Cox proportional hazards regression models were used to evaluate the association between consolidation intensity and overall survival while adjusting for relevant clinical factors, including transplant status. Hazard ratios (HRs) and 95% confidence intervals (CIs) were reported. A two-sided *p*-value <0.05 was considered statistically significant. All statistical analyses were conducted using SAS version 9.4 (SAS Institute, Cary, NC, USA).

## 3. Results

### 3.1. Patient Characteristics

We identified 129 patients aged ≥60 years out of which 111 patients met eligibility criteria for the 90-day landmark analysis (Table 1). The median age of the cohort was 65 years (range 60–75 years), 56% were male, and 77% were non-Hispanic White. According to ELN 2022 risk classification, 33% had favorable-risk AML, 27% intermediate-risk, and 40% adverse-risk disease. Sixteen (14%) patients had *FLT3*-mutated AML out of which nine patients received a *FLT3* inhibitor. Seven patients did not receive a *FLT3* inhibitor as they were treated prior to the FDA approval of those drugs. No other patients received additional agents. Most patients (86%) had *de novo* AML. All patients received standard 7 + 3 induction therapy, with idarubicin 12 mg/m^2^ administered in 53% and daunorubicin 45–60 mg/m^2^ in 47%, and eleven patients required re-induction prior to consolidation. The cytarabine dose during induction was 100 mg/m^2^. All patients were in first complete remission at the time of initiating cytarabine consolidation. The median follow-up for the cohort was 18 months (range 9–142 months).

### 3.2. Consolidation Therapy

Cytarabine consolidation was administered according to institutional practice, with 44% of patients receiving a condensed schedule (days 1, 2, and 3) and 56% receiving a standard schedule (days 1, 3, and 5). The median number of consolidation cycles administered was 2 (range 1–4); 20 (18%) patients received one cycle, 37 (33%) two cycles, 51 (46%) three cycles, and 3 (3%) four cycles. Five patients received 3 g/m^2^ per dose per cycle for 3 cycles. The cumulative median cytarabine dose across all cycles was 18 g/m^2^ (range 6–54 g/m^2^). Based on this median, 25 patients were categorized as receiving low-intensity consolidation (LIC, <18 g/m^2^), and 86 patients received high-intensity consolidation (HIC, ≥18 g/m^2^). Overall, 41% of patients proceeded to allo-SCT, and among non-transplanted patients, the median number of consolidation cycles was three (range 1–4).

### 3.3. Safety

Cytarabine consolidation was generally well tolerated. The median time to neutrophil recovery was 17 vs. 19 days and platelet recovery was 20 vs. 22 days in condensed and standard consolidation schedule, respectively. One patient experienced nephrotoxicity during the first cycle, which resolved with supportive care, and subsequently completed additional cycles. No cases of neurotoxicity were observed, indicating that both condensed and standard consolidation schedules were safe in this older population.

### 3.4. Survival Outcomes

**Figure 1:**
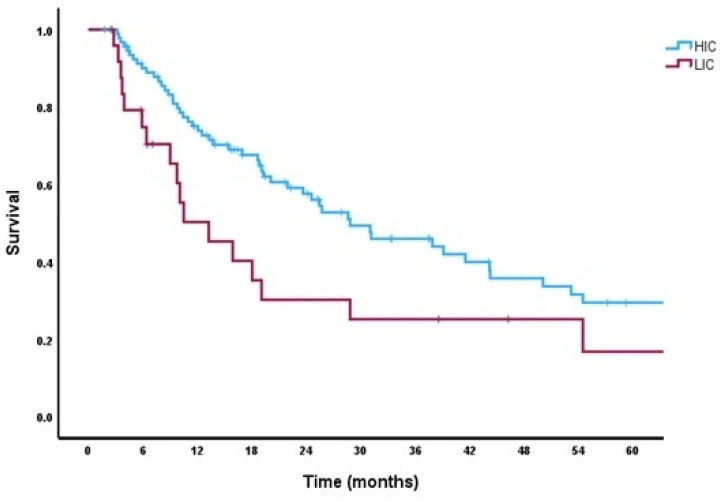
Overall survival by consolidation intensity.

Fourteen patients (56%) in the LIC group and 47 patients (55%) in the HIC groups relapsed, respectively. The median overall survival (mOS) and relapse-free survival (mRFS) for the entire population was 25 m and 19 m, respectively. The mOS for LIC vs. HIC was 13 m vs. 31 m (*p* = 0.02) (Figure 1). In patients that did not proceed to allo-SCT, the mOS for LIC vs. HIC was 7 m vs. 25 m (*p* = 0.01) (Supplementary Figure S4). The mOS was not statistically different between the two intensities for patients that proceeded to allo-SCT (Supplementary Figure S2). The mOS for allo-SCT vs. no allo-SCT was 39 m vs. 23 m (*p* = 0.05). The mOS for 1 vs. 3 cycles of cytarabine consolidation was 20 m vs. 31 m (*p* = 0.1) in all patients and 10 m vs. 26 m (*p* = 0.03) in non-transplanted patients. The mRFS for LIC vs. HIC was 10 m vs. 23 m (*p* = 0.1) (Supplementary Figure S1). The mRFS by consolidation intensity for transplanted and non-transplanted patients was not statistically different (Supplementary Figures S3 and S5). In multivariable Cox proportional hazards analysis adjusting for age, gender, disease risk stratification, consolidation intensity, consolidation schedule and transplant status, both HIC (HR = 0.71, 95% CI 0.51–0.82, *p* = 0.01) and allo-SCT (HR = 0.58, 95% CI 0.44–0.79, *p* = 0.03) were independently associated with improved overall survival.

## 4. Discussion

In our analysis, we report that a higher cumulative dose of cytarabine consolidation is safe, feasible, and associated with improved survival outcomes in older adults (≥60 years) with AML. Our cohort of 111 patients had a median age of 65 years, was well balanced across ELN 2022 risk categories, and included a mix of condensed and standard consolidation schedules. We used a cutoff of 18 g/m^2^ cumulative cytarabine to stratify patients into LIC and HIC groups based on the median cumulative dose and only included those reaching the 90-day mark from diagnosis to minimize the bias of early death and relapse.

A key finding of our study is the overall safety of cytarabine consolidation in older adults. Both LIC and HIC strategies were well tolerated regardless of schedule, with individual doses ranging mainly from 1 g/m^2^ to 2 g/m^2^. Most patients were able to receive two or more cycles of consolidation, demonstrating feasibility. Only one patient developed nephrotoxicity, which resolved with supportive care, and no neurotoxicity events were observed. These findings contrast with earlier studies, such as CALGB 8525, which reported high rates of toxicity in older patients receiving mainly receiving 3 g/m^2^ [2,3]. Our results align with more recent reports supporting the tolerability and efficacy of intermediate- or high-dose cytarabine in older adults where most of the patients received 1.5 g/m^2^ [6,13].

We found that higher cumulative cytarabine doses were associated with significantly improved overall survival. The difference was more pronounced among patients who did not proceed to allo-SCT. These findings suggest that higher cumulative doses may be particularly important in older patients who are not transplant candidates, providing effective post-remission disease control. In contrast, among patients who underwent allo-SCT, no significant difference in survival was observed between HIC and LIC, consistent with the concept that consolidation primarily serves as a bridge to transplantation in this population [14]. Our findings are in line with prior studies in older adults. Hassanein et al. reported a median OS of 25.6 months in patients ≥ 60 years receiving two cycles of 1.5 g/m^2^ cytarabine, corresponding to our HIC group [13]. Similarly, a study of 149 Chinese patients aged ≥60 demonstrated favorable outcomes and tolerability with intermediate- to high-dose cytarabine (1.5–2 g/m^2^) compared to multi-agent consolidation regimens [6]. Nearly half the patients in our study received a condensed consolidation schedule with treatment on days 1–3. We found no difference in safety and efficacy based on condensed scheduling. More recently, others have also demonstrated the feasibility of administrating cytarabine consolidation in a condensed schedule [15]. However, all these studies share limitations inherent to retrospective designs, including potential selection bias for fitter patients able to tolerate intensive therapy [16]. It is worth noting that HIC was associated with improved OS and a numerical but not significant improvement in RFS. Although the exact etiology for this remains unclear, a possibility may be that receipt of HIC is likely a surrogate for variables that could make patients tolerate post-relapse therapy better.

Compared to older patients, the optimal consolidation dose of cytarabine in younger adults has been studied extensively. Initial studies established 3 g/m^2^ as the standard dose but more recently Hunault et al. found non-inferiority of 1.5 g/m^2^ to 3 g/m^2^ in adults aged 18–60 [5,17,18]. However, it is worth noting that 71.8% of the patients in the study proceeded to allo-SCT and that whether there was a difference in outcomes in non-transplanted patients is not clear. Only 5 patients in our study received 3 g/m^2^ and nearly 60% did not proceed to allo-SCT. Our data suggest that older adults, particularly those ineligible for transplantation, may still derive meaningful benefit from higher cumulative cytarabine doses.

This study has several limitations. It is a single-center, retrospective analysis, which introduces the potential for selection bias and unmeasured confounding inherent to such studies. In clinical practice, physicians may preferentially administer lower cumulative doses of cytarabine to patients perceived to be less fit because of comorbidities, frailty, poorer performance status, or concerns regarding treatment-related toxicity. Consequently, the superior survival outcomes observed in the HIC group may partly reflect differences in baseline patient fitness rather than the independent effect of consolidation intensity alone. Although patients included in this study were considered eligible for intensive induction and consolidation therapy based on institutional practice standards, detailed standardized comorbidity indices, frailty assessments, and geriatric evaluation data were not consistently available for retrospective collection and therefore could not be fully incorporated into the analysis. Prospective studies incorporating formal fitness and comorbidity assessments will be important to better define the independent contribution of cumulative cytarabine dose intensity in older adults with AML [19,20,21]. Additionally, heterogeneity in consolidation schedules and the number of cycles, though reflective of real-world practice, may reduce generalizability. A further limitation is the lack of standardized measurable residual disease (MRD) assessment [22]. Due to the retrospective design and long study period (2012–2024), MRD testing was not uniformly performed, and data were not consistently available for analysis. Therefore, we were unable to evaluate the impact of consolidation intensity on MRD clearance or incorporate MRD status into outcome analyses. Prospective studies with standardized MRD assessment are needed to clarify the relationship between cytarabine exposure, depth of remission, and survival in older adults with AML. Furthermore, with the uptake of less-intensive chemotherapy regimens in the older patients with AML, the role of intensive chemotherapy is being increasingly questioned [23,24,25]. However, our data shows that a proportion of older patients are indeed candidates for intensive chemotherapy. Lastly, induction with a less-intense regimen followed by consolidation with cytarabine may be a strategy for some patients. Nonetheless, our findings provide important insight into post-remission consolidation strategies in a population with limited prospective data.

## 5. Conclusions

In conclusion, our study demonstrates that a higher cumulative dose of cytarabine consolidation is safe, feasible, and associated with improved survival in older adults with AML, particularly among patients who do not undergo allo-SCT. These findings support reconsideration of consolidation dosing strategies in this population and highlight the need for prospective studies to define the optimal cytarabine consolidation approach for older adults.

## Figures and Tables

**Table 1:** Baseline characteristics.

	LIC (n = 25)	HIC (n = 86)	*p*-Value	Total (N = 111)
**Median age, years (range)**	64 (60–74)	65 (60–75)	0.9	65 (60–75)
**Male, n (%)**	14 (56%)	48 (56%)	1	62 (56%)
**Non-Hispanic White, n (%)**	19 (76%)	65 (76%)	1	84 (77%)
**ELN 2022 risk, n (%)**			0.8	
- Favorable	8 (32%)	29 (34%)		37 (33%)
- Intermediate	7 (28%)	23 (27%)		30 (27%)
- Adverse	10 (40%)	34 (40%)		44 (40%)
**De novo AML, n (%)**	21 (84%)	74 (86%)	0.8	95 (86%)
**Anthracycline: idarubicin, n (%)**	13 (52%)	46 (53%)	0.9	59 (53%)
**Anthracycline: daunorubicin, n (%)**	12 (48%)	40 (47%)	0.9	52 (47%)
**Re-induction required, n (%)**	2 (8%)	9 (10%)	0.8	11 (10%)
**Consolidation schedule, n (%)**			1	
Condensed (days 1–3)	11 (44%)	38 (44%)		49 (44%)
Standard (days 1, 3, 5)	14 (56%)	48 (56%)		62 (56%)
**Median number of cycles (range)**	2 (1–3)	2 (1–4)	1	2 (1–4)
**Median cumulative dose, g/m^2^ (range)**	12 (6–17)	24 (18–54)	N/A	18 (6–54)
**Allo-SCT, n (%)**	10 (40%)	35 (41%)	0.8	45 (41%)

## Data Availability

The data that support the findings of this study are available on request from the corresponding author.

## Supplementary Materials

The following supporting information can be downloaded at: https://www.mdpi.com/article/10.3390/cancers18111831/s1, Figure S1: Relapse-free survival by consolidation intensity; Figure S2: Overall Survival by consolidation intensity in transplanted patients; Figure S3: Relapse-free Survival by consolidation intensity in transplanted patients; Figure S4: Overall Survival by consolidation intensity in non-transplanted patients; Figure S5: Relapse-free Survival by consolidation intensity in non-transplanted patients.
