# Supplementary material for: Higher Cumulative Cytarabine Consolidation Improves Survival in Older Adults with Acute Myeloid Leukemia
Source: Cancers (Basel). 2026 Jun 3;18(11):1831. doi: 10.3390/cancers18111831 (PMC13256100; doi:10.3390/cancers18111831)

Supplementary Figure S1: Relapse-free survival by consolidation intensity

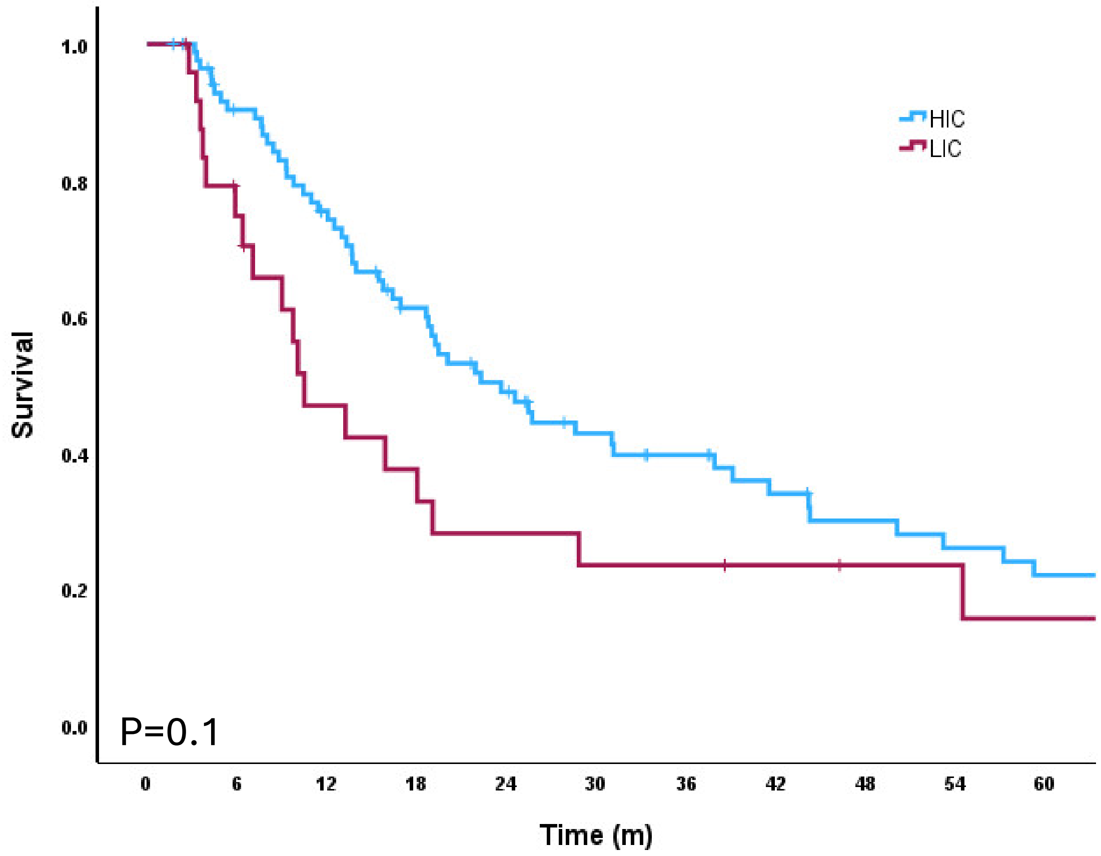

Supplementary Figure S2: Overall Survival by consolidation intensity in transplanted patients

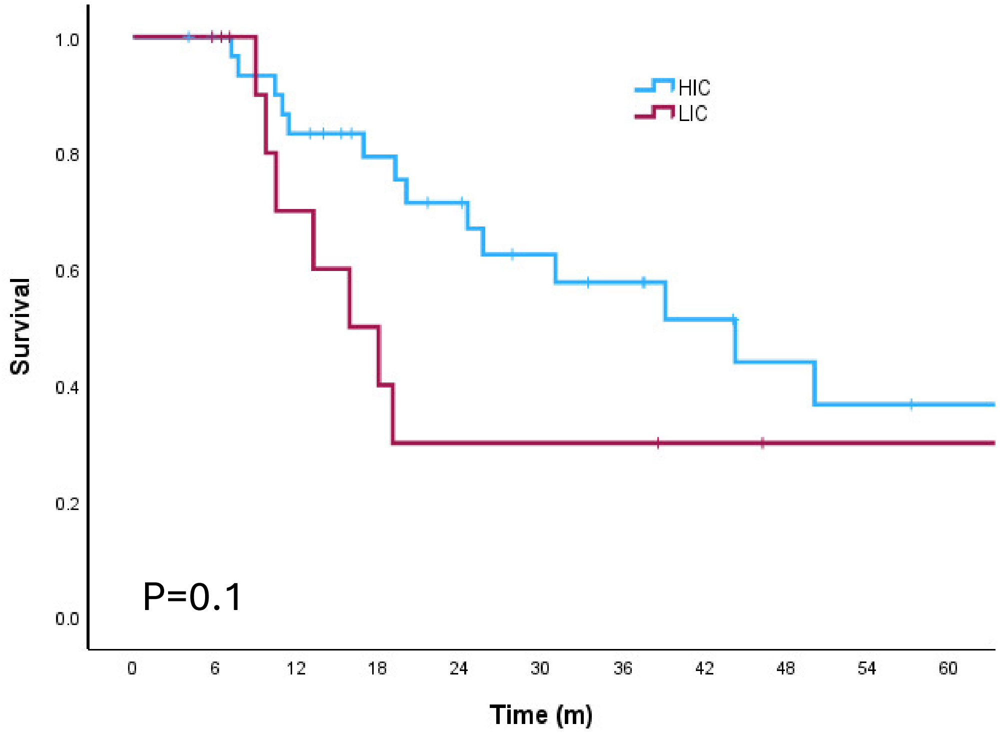

Supplementary Figure S3: Relapse-free Survival by consolidation intensity in transplanted patients

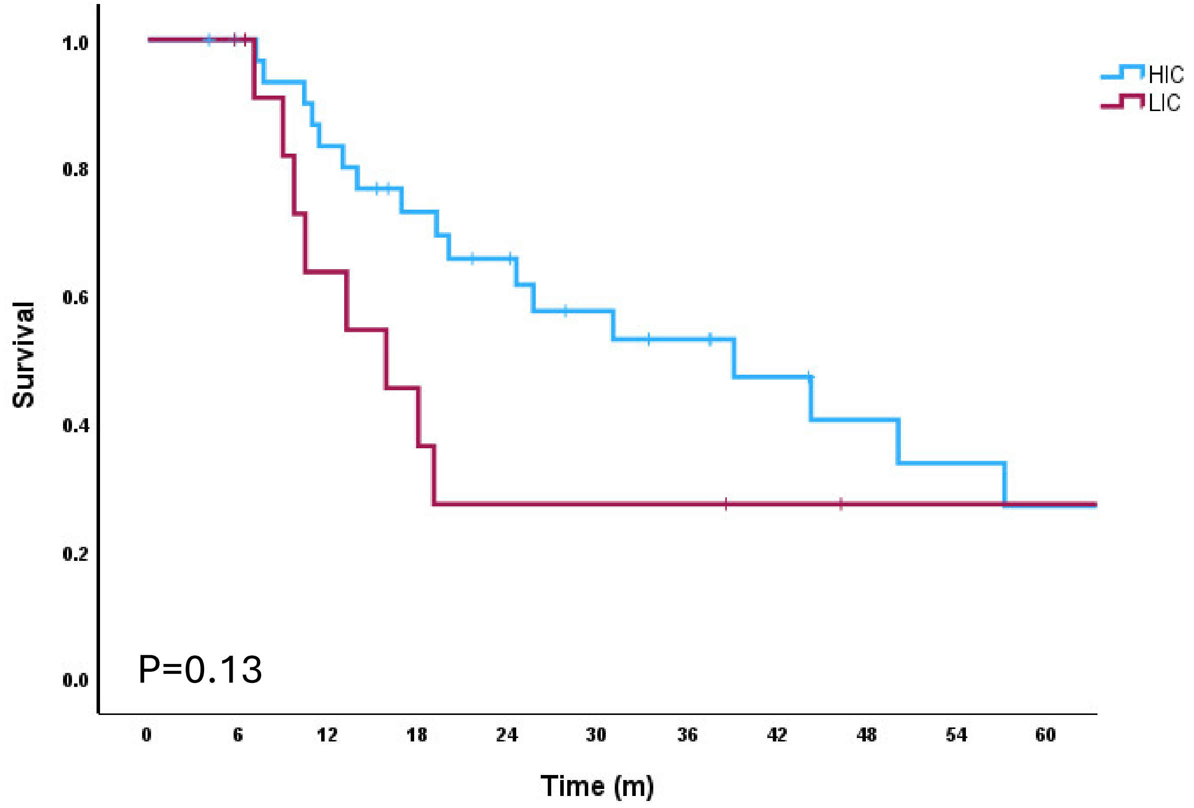

Supplementary Figure S4: Overall Survival by consolidation intensity in non-transplanted patients

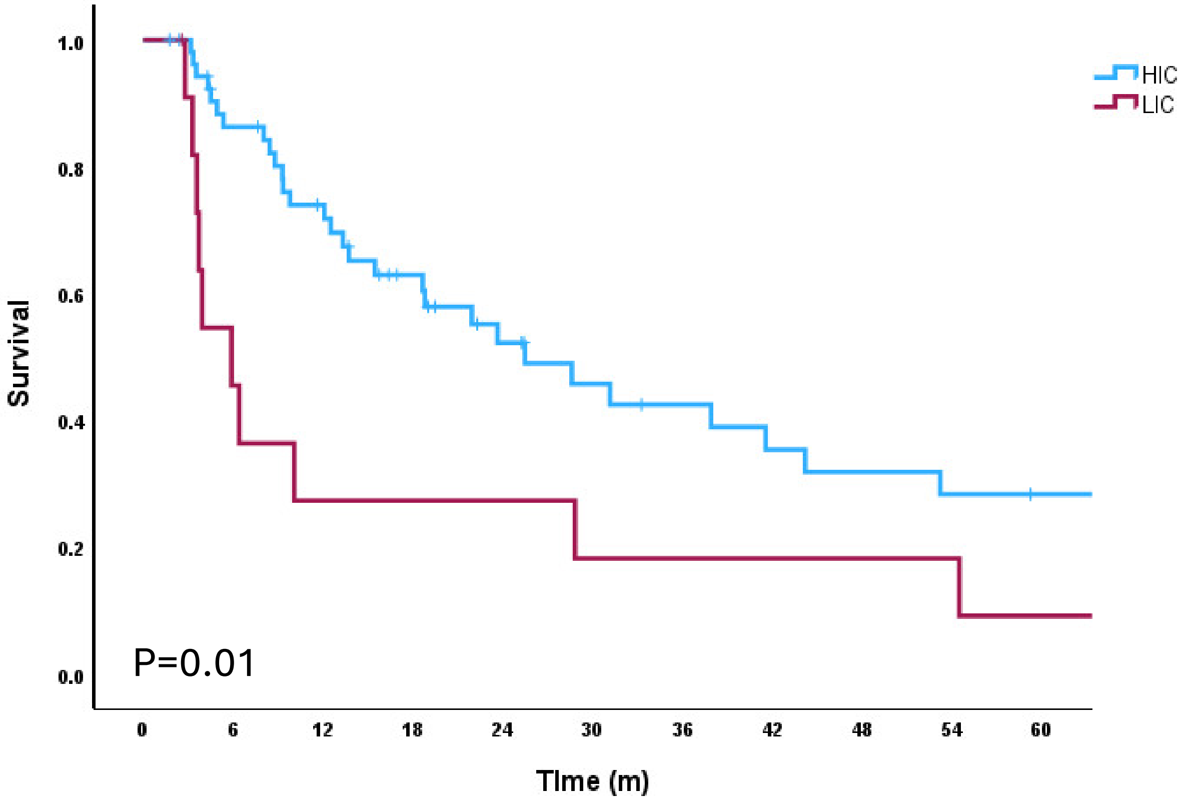

Supplementary Figure S5: Relapse-free Survival by consolidation intensity in non-transplanted patients

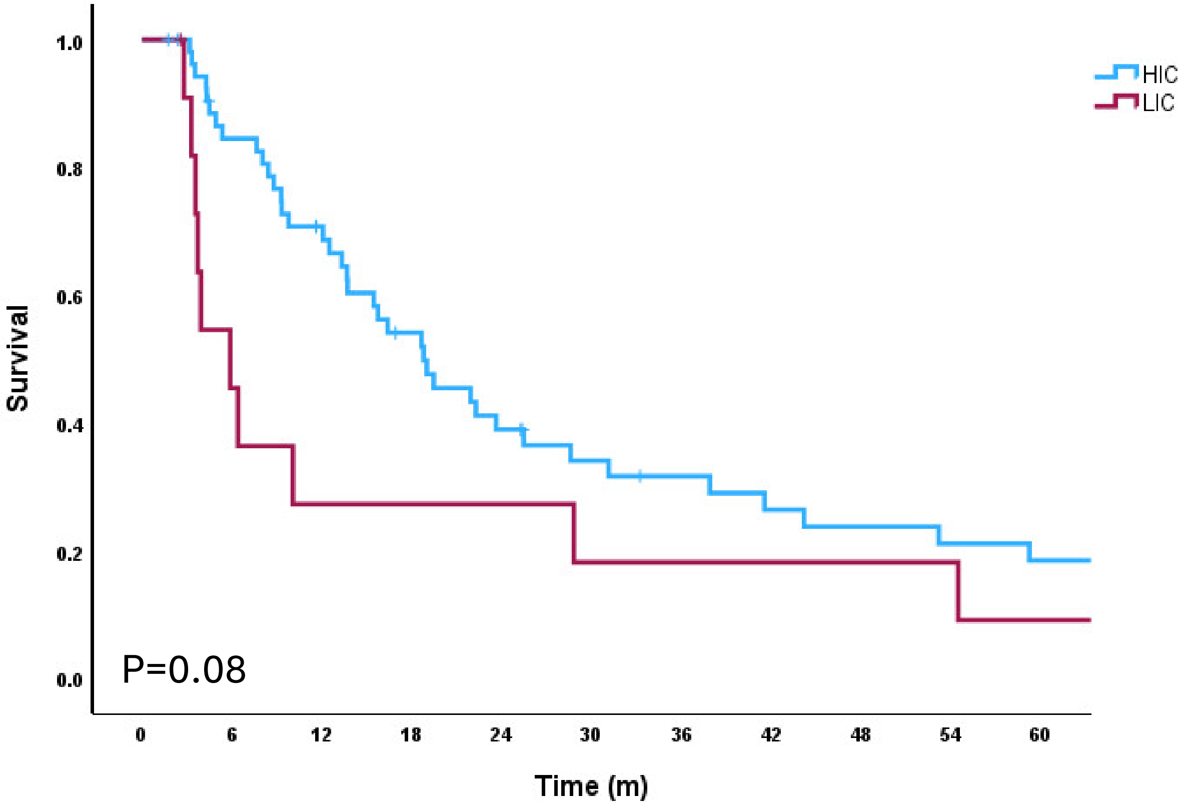

Supplement: Supplementary file 1 [file cancers-18-01831-s001.zip › cancers-4317704-supplementary.pdf]
